# The 100 most-cited articles on aortic dissection

**DOI:** 10.1186/s12872-016-0426-9

**Published:** 2017-01-17

**Authors:** Ping Lai, Yuan-hui Liu, Jin-hua Xue, Peng-cheng He, Yue-qun Qiu

**Affiliations:** 1 Medical Student, Gannan Medical University, Ganzhou, 341000 China; 2 Department of Cardiology, Guangdong Cardiovascular Institute, Guangdong Provincial Key Laboratory of Coronary Heart Disease Prevention, Guangdong General Hospital, Guangdong Academic of Medical Sciences, Guangzhou, 510080 China; 3 Department of Pathophysiology, School of Basic Medical Sciences, Southern Medical University, Guangzhou, 510515 China; 4 Department of Physiology, School of Basic Medical Sciences, Gannan Medical University, Ganzhou, 341000 China; 5 Yuequn Qiu, MS, Department of Cardiology, Ganzhou cardiology center, Society for Cardiovascular Internal Medicine, Emergency Medicine, The First Affiliated Hospital of Gannan Medical University, Ganzhou, 341000 China

**Keywords:** Most-cited, Aortic dissection

## Abstract

**Background:**

To identify and characterize the most frequently cited articles that have been published on aortic dissection.

**Methods:**

A list of the 100 most frequently cited publications (T100) about aortic dissection was generated by performing a searching of the Science Citation Index--Expanded using “aortic dissection” as the search term. Basic information about the articles was recorded, including number of citations, journal title, journal impact factor, time since publication, first author’s country, topic/subspecialty of the research, and publication type.

**Results:**

We finally included 180 articles on aortic dissection, from which we identified the 100 most frequently cited articles (T100). The most frequently cited article received 1079 citations, while the least frequently cited article received 68 (mean140.5 citations per article). The T100 originated from 19 countries, with more than half of them originating from the USA (*n* = 97). The T100 articles were published from 1955 to 2013, with 79% published during the period 1990–2009. In addition, there were 40 different journals with Circulation having the most citations (*n* = 38). Regarding the article type, there were 21 basic and 140 clinical research articles, one meta-analysis, and 18 review articles. Reviews had the highest mean number of citations (mean 235.5 citations per article).

**Conclusions:**

Our study provides a historical perspective on the progress of dissection research, and helps to identify the quality of the work, the discoveries made, and the trends steering the studies.

**Electronic supplementary material:**

The online version of this article (doi:10.1186/s12872-016-0426-9) contains supplementary material, which is available to authorized users.

## Background

Aortic dissection (AD) is a life-threatening condition, with 4.2% of patients dying in hospital as a result of the condition, and its prompt diagnosis remains essential for successful management. Large numbers of articles about AD have been published annually, and these have given new insights into its mechanism, diagnosis and treatment.

Citation analysis is an important tool to apprise past research advances comprehensively and to predict future research trends in a specific field. Although it is unwise to assess the quality of a study based only on its citation rating, the frequency of citing has significant implications for authors, journals, institutions and even countries, as it is one of the markers of the impact of an individual scholarly work within the scientific community [1]. Therefore, various techniques have been used to identify the most frequently cited articles in both medical and surgical specialties, including traumatic brain injury [2], neurosurgery [3], orthopedics [4], cardiac surgery [5], urology [6], cardiovascular disease [7], hypertension [8], and lung disease [9].

AD has experienced an epidemiological transition, with mortality due to dissections, such as stroke and bleeding episodes, being up to 5% per year [10]. In recent decades, there has been great progress in AD research worldwide, and the condition has gained greater attention from the world community. However, there is a lack of articles assessing the quality of the scientific yield in this area. Therefore, this study aimed to analyze the characteristics of the 100 most-frequently cited articles published about dissection, and to provide information about the achievements and development that have occurred in AD research.

## Methods

We used the Science Citation Index Expanded from ISI Web of Science (Thomson Reuters, Philadelphia, PA, USA) to obtain citation information about published articles on AD. No time limitations were implemented for the investigation, and there were no restrictions on study types, abstract availability, or human versus nonhuman studies. Using the search term “aortic dissection” identified more than 1000 articles. From these, we selected 180 articles, and these were used to identify the 100 most-cited articles in our study. We included all articles with the same citations. Therefore, there were 180 articles that were used to identify the 100 most-cited articles in our study. All of these articles were sorted by the number of times cited, which provided us with a list of all the articles published in a specific journal ranked by citation count. The top 100 articles according to the number of citations (T100) were analyzed by two independent reviewers who used a modified approach of the methods published by Lim et al. [11].

Information on the journal name, title, journal impact factor(IF), paper title, number of citations, decade in which the paper was published, publication year, number of authors, country of origin, and topic/subspecialty of the research was recorded. In addition, the study types (meta-analysis, basic research, or clinical research were determined by another two independent reviewers, based on the titles and abstracts. Disagreements were resolved by discuss on. Associations between the journal IF and the number of articles, and between the journal IF and the number of its papers included in the T100 articles list were assessed using the 2014 edition of Journal Citation Reports (JCR): Science Edition (1945–2013).

### Statistical analysis

Data were expressed as median and interquartile range. The Wilcoxon rank sum test was performed to evaluate differences between groups. The Spearman test was used to evaluate the strength and direction of the linear relation between the IF of the journal and the numbers of citations or T100 articles. All data analyses were performed using SAS software, version 9.2 (SAS Institute, Cary, NC, USA). All probability values were two-tailed, and statistical significance was defined as *p* < 0.05.

## Results

**Fig. 1:**
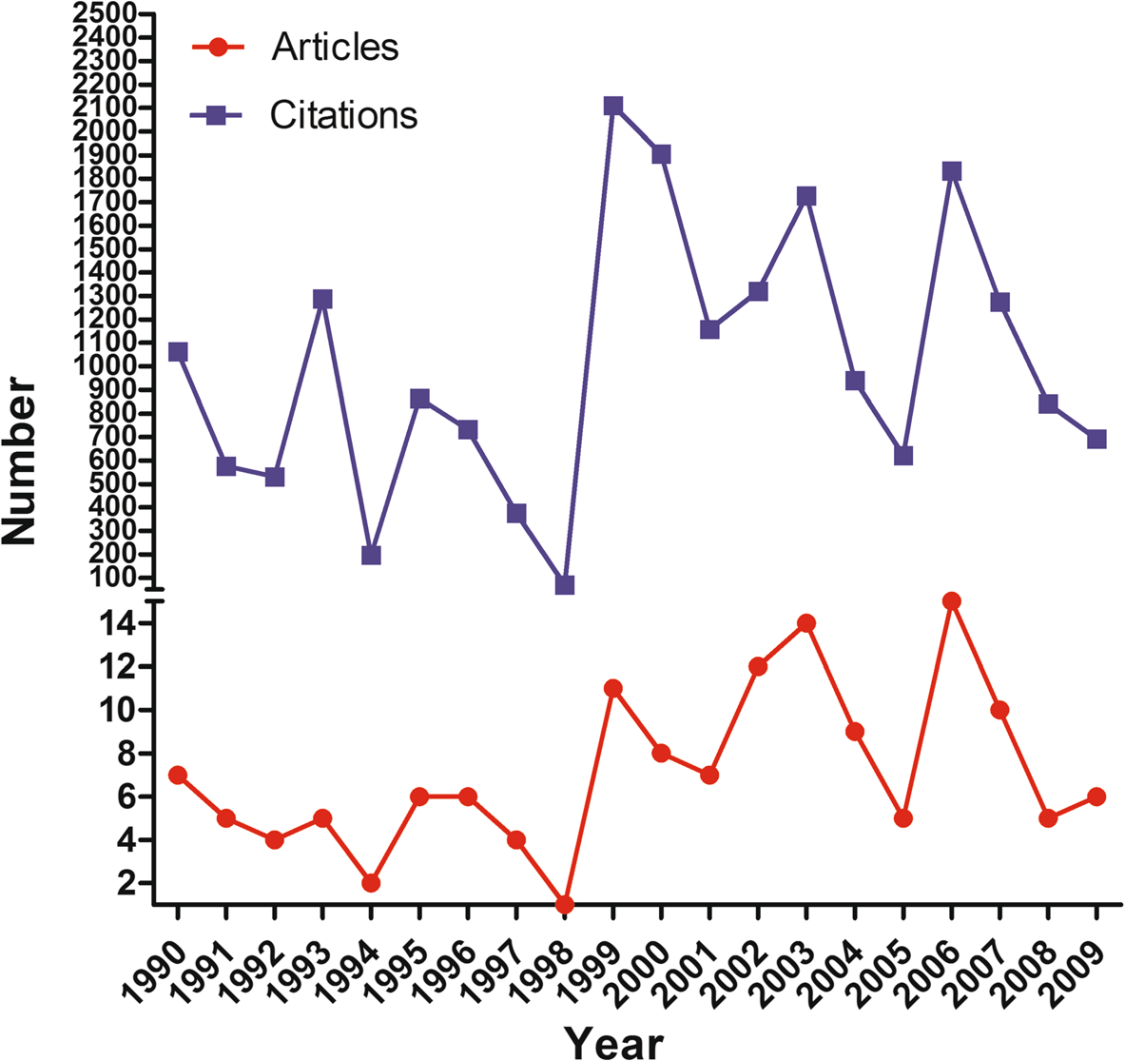
Numbers of the most frequently cited articles published from 1990 to 2009

The mean number of citations for the T100 articles was 140.8 (range 68–1079), Only five papers were cited over 500 times, and only one of those papers was published in the latest 3 years (2013–2015). In the T100 list 79% of the papers were published during the period 1990–2009 (Fig. 1), and the mean number of citations per year during that 20-year period was 1005.8 (range 69–2111). The T100 articles are listed in Table 1 and Additional file 1 (1–20 in Table 1; with the remainder of the list (21–100) given in Additional file 1 as supplementary data on-line).

**Table 1:** Bibliometric information associated with the Top 20 of the Top 100 most frequently cited articles (T100) on dissection

Rank	Author	Title	Journal	Year	Times cited	PMID
1	Hagan, PG; et al..	The International Registry of Acute Aortic Dissection (IRAD) - New insights into an old disease	Jama-Journal of The American Medical Association	2000	1079	10685714
2	Dake, MD;et al.	Endovascular stent-graft placement for the treatment of acute aortic dissection	New England Journal of Medicine	1999	658	10332016
3	Nienaber, CA;et al.	Nonsurgical reconstruction of thoracic aortic dissection by stent-graft placement	New England Journal of Medicine	1999	591	10332015
4	Erbel, R; et al.	Diagnosis and management of aortic dissection - Recommendations of the Task Force on Aortic Dissection, European Society of Cardiology	European Heart Journal	2001	522	11511117
5	Nienaber, CA; et al.	The diagnosis of thoracic aortic dissection by noninvasive imaging procedures	New England Journal of Medicine	1993	504	8416265
6	Larson, EW; et al.	Risk-factors for aortic dissection - a necropsy study of 161 cases	American Journal Of Cardiology	1984	441	6702637
7	Erbel, R; et al.	Echocardiography in diagnosis of aortic dissection	Lancet	1989	394	2563839
8	Sasako, Mitsuru; et al.	D2 lymphadenectomy alone or with para-aortic nodal dissection for gastric cancer	New England Journal of Medicine	2008	339	18669424
9	Roberts, WC	Aortic dissection - anatomy, consequences, and causes	American Heart Journal	1981	310	7008565
10	Evans, JM; et al.	Increased incidence of aortic-aneurysm and dissection in giant-cell (temporal) arteritis - a population-based study	Annals of Internal Medicine	1995	288	7872584
11	Meszaros, I; et al.	Epidemiology and clinicopathology of aortic dissection - A population-based longitudinal study over 27 years	Chest	2000	278	10807810
12	Eggebrecht, H; et al.	Endovascular stent-graft placement in aortic dissection: a meta-analysis	European Heart Journal	2006	273	16227309
13	Desanctis, RW;et al.	Aortic dissection	New England Journal Of Medicine	1987	271	3309654
14	Guo, Dong-Chuan; et al.	Mutations in smooth muscle alpha-actin (ACTA2) lead to thoracic aortic aneurysms and dissections	Nature Genetics	2007	262	17994018
15	Anagnost.CE; et al.	Aortic dissections and dissecting aneurysms	American Journal of Cardiology	1972	253	4557973
16	Cigarroa, JE; et al.	Diagnostic-imaging in the evaluation of suspected aortic dissection - old standards and new directions	New England Journal of Medicine	1993	242	8416269
16	Miller, DC; et al.	Operative treatment of aortic dissections - experience with 125 patients over a 16-year period	Journal of Thoracic And Cardiovascular Surgery	1979	242	470417
17	Ueda, Y;et al.	Surgical-treatment of aneurysm or dissection involving the ascending aorta and aortic-arch, utilizing circulatory arrest and retrograde cerebral perfusion	Journal of Cardiovascular Surgery	1990	239	2229147
18	Spittell, PC; et al.	Clinical-features and differential-diagnosis ofaortic dissection - experience with 236 cases (1980 through 1990)	Mayo Clinic Proceedings	1993	233	8350637
19	Erbel, R; et al.	Effect of medical and surgical therapy on aortic dissection evaluated by transesophageal echocardiography - implications for prognosis and therapy	Circulation	1993	228	8491016
20	Nienaber, Christoph A.; et al.	Randomized Comparison of Strategies for Type B Aortic Dissection The INvestigation of STEnt Grafts in Aortic Dissection (INSTEAD) Trial	Circulation	2009	222	19996018

**Fig. 2:**
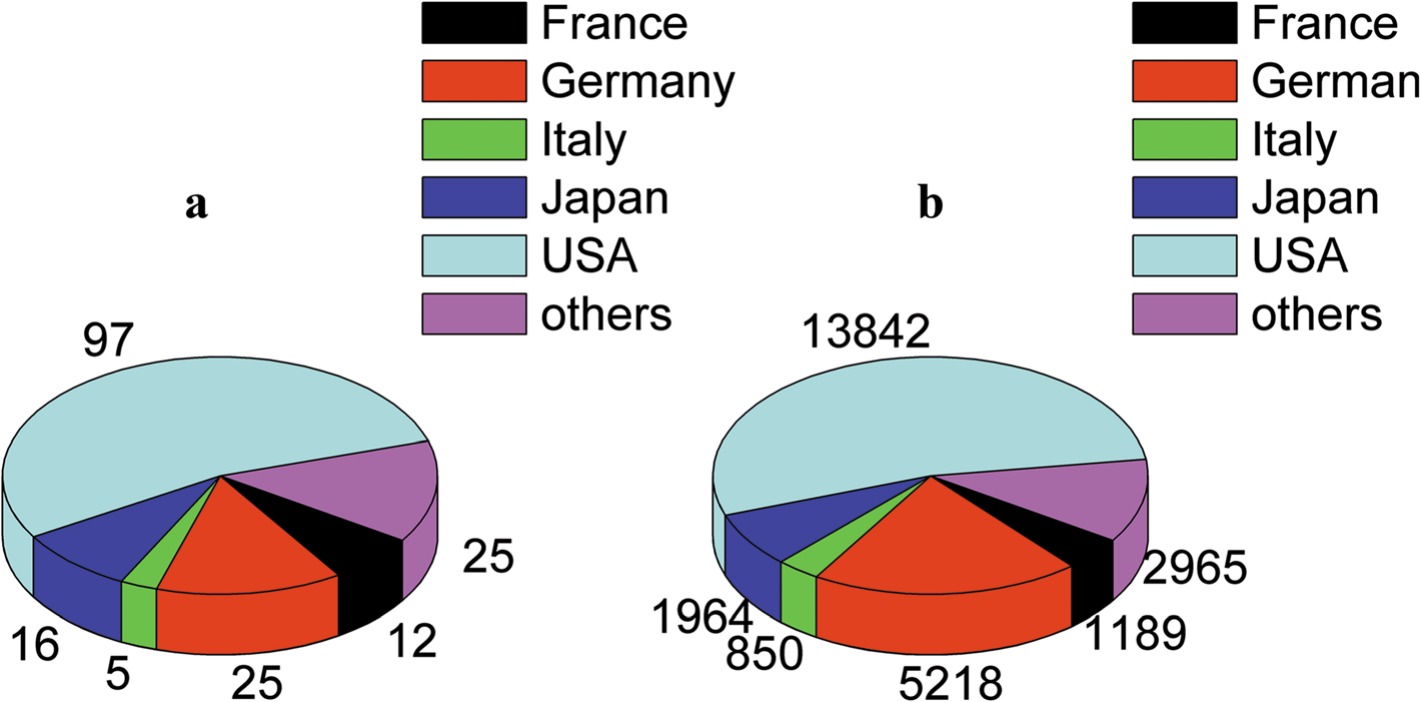
The T100 were analyzed in terms of their country of origin. **a** Number of articles from each country. **b** Number of citations for the articles from each country

Based on the address of the corresponding author, we found that authors from 19 different countries had contributed to the T100 articles, with the USA being the top contributor (*n* = 97), followed by Germany (*n* = 25), Japan (*n* = 16), France (*n* = 12), and Italy (*n* = 5), while the remaining countries had <5 highly cited articles (Fig. 2a-b). However, the articles with the highest mean number of citations per article (208.7) originated from Germany.

For most of the classic articles, the first author was affiliated with an academic department. Most of the authors had only one highly cited article among the T100 articles, and only one author had 10 highly cited articles as the first author, with a mean of 222 citations. The five authors with the most citations had 1079, 658, 591,522, and 441 citations, respectively.

**Fig. 3:**
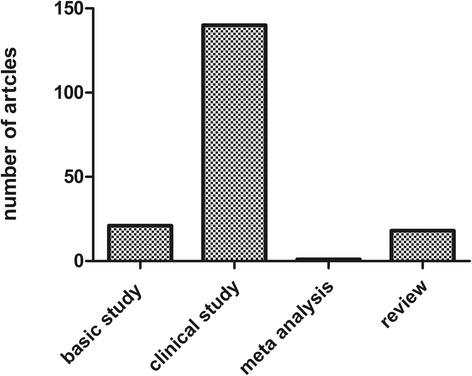
Publication type distributions for the T100

**Fig. 4:**
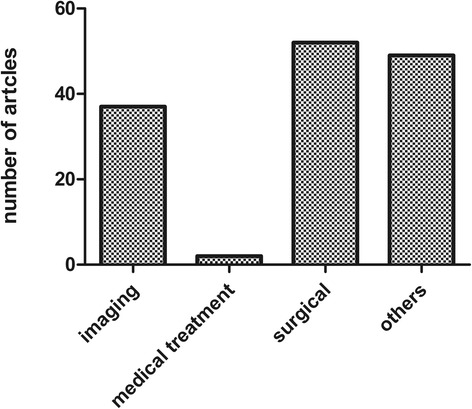
Research topic distributions for the clinical articles

**Fig. 5:**
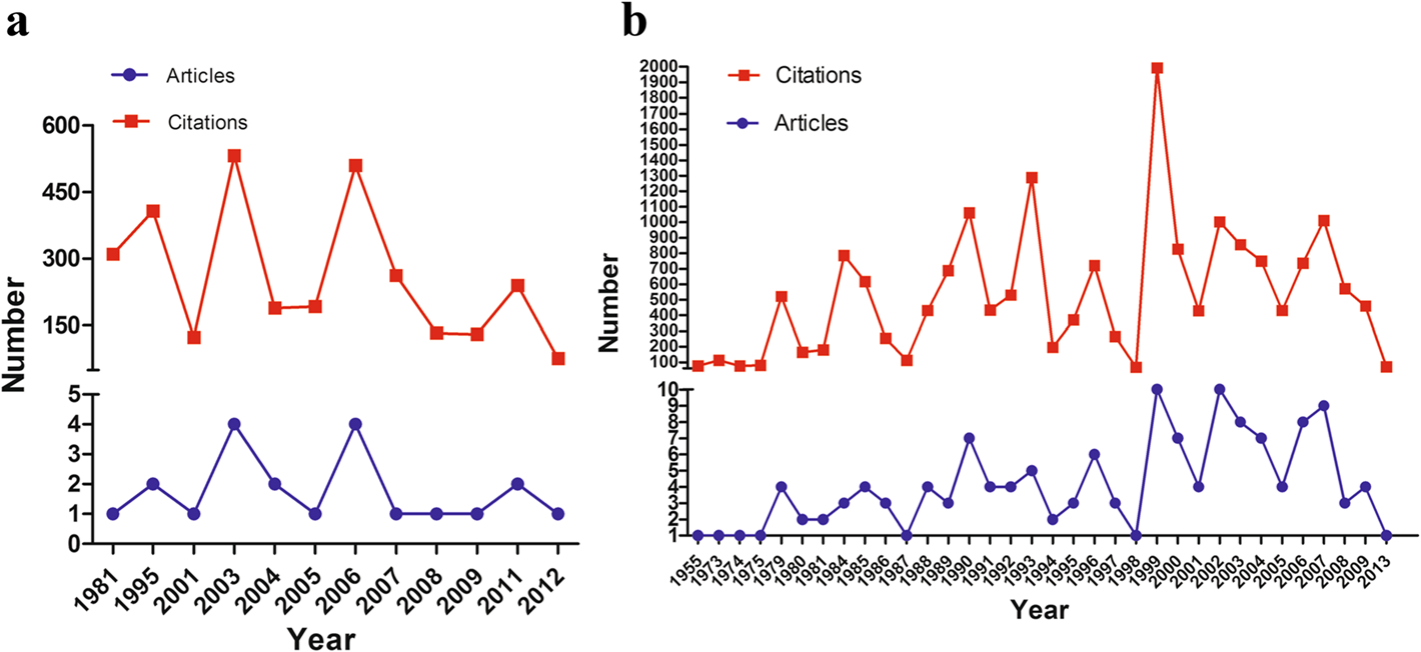
Basic studies and clinical studies were analyzed in terms of their publication time. **a** Number of basic articles and number of citations for these basic articles in each year. **b** Number of clinical articles and number of citations for these clinical articles in each year

The T100 articles were published in 40 different journals, predominantly in Circulation (*n* = 38), Journal of Thoracic and Cardiovascular Surgery (*n* = 19), Annals of Thoracic Surgery (*n* = 18), Radiology (*n* = 14), Journal of the American College of Cardiology (*n* = 10), Journal of Vascular Surgery (*n* = 9), and New England Journal of Medicine (*n* = 7). The IF of the journals that published the T100 articles ranged from 1.4 to 55.8. However, the three leading medical journals, which have extremely high IF values, contributed only 11 articles to the list with the Journal of the American Medical Association contributing none, while The Lancet and New England Journal of Medicine contributed four and seven, respectively. Many of the T100 articles were published in high IF journals (journals in which the T100 articles were published are shown in Table 2); however, the journal IF did not correlate with the number of T100 articles, but did correlate with the number of citations (*r* = 0.38, *p* = 0.017). For the T100 articles, the majority of the article types were clinical trials (*n* = 140), followed by basic science studies (*n* = 21), reviews (*n* = 18), and meta-analyses (*n* = 1) (Fig. 3). In the basic research articles, more tended to molecular studies (such as tumor necrosis beta2, interleukin and so on), whereas before 2005, most of the articles were anatomical or cell (eg. giant cells) research. Few gene studies were included in the basic research articles. Of the 140 clinical articles, 54 were related to surgery and 35 to imaging (computed tomography/magnetic resonance imaging or ultrasound), 49 articles were related to other topics. As shown in Fig. 4, the articles relating to surgery were focused on grafting (16 studies), endovascular repair (6 studies), biological glue (3 studies), new skills (2 studies), or other topics (18 studies). Comparing the basic science articles with clinical studies with regard to year of publication, we found that the clinical studies were on average published earlier than the basic science studies with only three of the most frequently cited basic articles published before 2000 (239 times per article), whereas the three most frequently cited clinical studies were all published in 1984 (262.3 times per article) (Fig. 5a-b). Regarding total citations, clinical articles ranged from 68 to 658 (mean 130.0), while basic science articles ranged from 73 to 310 (mean 147.8).

**Table 2:** Journals in which the T100 were published

Journal	No. of Articles (Citations)	Impact Factor
Circulation	38 (5126)	14.43
Journal of Thoracic and Cardiovascular Surgery	19 (2095)	4.168
Annals of Thoracic Surgery	18 (1682)	3.849
Radiology	14 (1498)	6.867
Journal of The American College of Cardiology	10 (1105)	16.503
Journal of Vascular Surgery	9 (837)	3.021
American Journal of Cardiology	7 (1196)	3.276
New England Journal of Medicine	7 (2808)	55.873
Nature Genetics	4 (713)	29.352
American Heart Journal	3 (533)	4.463
American Journal of Roentgenology	3 (223)	2.731
Annals of Surgery	3 (414)	8.327
Chest	3 (588)	7.483
European Heart Journal	3 (881)	15.203
Jama-Journal of The American Medical Association	3 (1296)	35.289
Lancet	3 (639)	45.217
Mayo Clinic Proceedings	3 (452)	6.262
Archives of Internal Medicine	2 (258)	17.333
Arthritis and Rheumatism	2 (269)	3.925
European Journal of Vascular And Endovascular Surgery	2 (191)	2.49
Journal of Cardiovascular Surgery	2 (312)	1.461
Journal of Computer Assisted Tomography	2 (255)	1.411
Journal of Endovascular Therapy	2 (179)	3.353
Radiographics	2 (150)	2.602
American Journal of Human Genetics	1 (119)	10.931
Annals of Internal Medicine	1 (288)	17.81
Annual Review of Genomics and Human Genetics	1 (133)	8.957
Arteriosclerosis Thrombosis and Vascular Biology	1 (170)	6
British Heart Journal	1 (114)	1.2
Cancer	1 (83)	4.889
Cardiology In The Young	1 (88)	0.835
Circulation Research	1 (81)	11.019
Circulation-Cardiovascular Interventions	1 (72)	6.218
Critical Care Medicine	1 (72)	6.312
European Journal of Cardio-Thoracic Surgery	1 (96)	3.304
Jacc-Cardiovascular Interventions	1 (147)	7.345
Journal of Clinical Investigation	1 (130)	13.215
Journal of Pediatrics	1 (115)	3.79
Medicine	1 (96)	5.723
Radiologic Clinics of North America	1 (70)	1.984

## Discussion

The present study assessed the characteristics of the 100 most frequently cited articles (T100) in the field of AD, which allowed us to uncover historical patterns and trends in the research about AD, which has undergone considerable change worldwide over the years. Furthermore, knowledge and understanding of the features inherent to frequently cited work should help young researchers to publish more effectively.

Citation analysis of articles in specific fields is important for both authors and journals; journals use it to attract better articles, while it provides researchers with some related advance information about diagnosis and therapy their research areas, which should help them produce better work. The number of articles labeled as “most cited” or “top cited” in various medical fields is increasing, but to date, there has been no citation analysis of articles about AD. Although the statistics are incomplete, it is likely that there are more than 180 000 patients with AD, and this value will continue to increase. The mortality rates as high as 50% without prompt treatment. However, reports concerning citation of articles on dissection are rare. In recent years, many studies have provided insight into the citation frequency of the most-cited articles published in many journals [12, 13], and therefore, analyzing the frequency of article citations in a condition such as AD, which has seen great advances and changes worldwide is necessary. This could help researchers recognize the quality of the research work, and identify the discoveries made, and could promote the development of treatments for AD.

Of the T100 cited articles, most were provided by authors from the USA provide the most (*n* = 97), followed by Germany (*n* = 25), confirming the important influence of both countries in relation to dissection diseases and research worldwide. This result is in concordance with many other studies. The reason for this may be that these two countries have a high burden of dissection disease and have sufficient financial support from the private or public sector to perform basic or clinical research as financial support from public foundations or commercial companies has evolved over time in response to changes in professional codes, laws, and markets [14].

It is well recognized that IF is important to both journals and authors, journals with high numbers of citations attract higher-quality articles, while authors who have written high-quality articles prefer to publish in high IF journals to get more attention. In the current study, we demonstrated that the number of citations were positively correlated with the IF, which is in accordance with our previous research [7]. Although it is unwise to assess the quality of a study based only on the citation rating, our study is consistent with the result of other reviews, in that most of the classic papers were published in high IF journals. The most frequently cited articles are often published in journals topping the IF list, which in turn maintains the high IF of these journals [15], and we found that 73 of these T100 cited articles were published in a high IF journal such as Circulation, Journal of the American College of Cardiology, New England Journal of Medicine, The Lancet, Nature Genetics, European Heart Journal, Archives of Internal Medicine, Annals of Internal Medicine, Circulation-Cardiovascular Interventions, and Journal of Clinical Investigation. However, there appears to be a more recent trend for authors to publishing highly influential articles in specialty journals rather than in general medical journals, and other bibliometric analyses have also demonstrated that highly influential reports are often published in specialty journals. Our study also found similar result in that some highly cited articles were published in journals with relatively low IF, such as Annals of Thoracic Surgery or Journal of Thoracic and Cardiovascular Surgery. (37 articles were published in these two journals, which had IFs of 3.8 and 4.1, respectively).

Time from publication also has an influence. Generally, a longer lapse from publication results in more citations, as each article has more time to be cited, which, it could explain why no articles published in 2014–2015 were on this list. However, this is not a complete explanation, as the most cited article in this study was published in 2000.

The high mortality associated with AD has attracted researchers to studying it. In the current study, we found that most of the T100 papers focused on clinical research. Stent implantation is the main method for the treatment of AD, and in accordance with this result, a large majority of the most highly cited clinical articles were focused on grafts. However, articles describing new methods such as biologic glue and endovascular repair were rare, with only nine related studies making the list. The prevalence of dissections is increasing dramatically (about 180 000 at present) and the high mortality from AD encourages recruitment of participants into research studies. Therefore, this analysis provides a direction for future research.

Our result with regard to the type of research report in the T100 was that clinical studies were much more common than basic studies, which is similar to the results of other analyses [16]. However, basic science research plays an important role in research into diseases, including AD. Therefore, physicians should design more basic science studies and collect basic data on AD. Several reasons contribute to the higher status of clinical research. First, AD is an emergency presentation, that needs urgent treatment, and many hospitals do not have sophisticated basic equipment to perform basic research. Second, insufficient funding is an important barrier to performing basic research, and many clinicians believe that they do not have enough time to perform basic science research along with patient care [17]. Much more basic research is needed to elucidate the mechanism of AD to allow better clinical treatment strategies, as several of the articles (Duggirala A et al. [18], Stankowski RV et al. [19] and Milewicz DM et al. [20]) in our T100 list, although published in journals with a relatively low IF provide important data about AD.

### Limitations

Our study has some limitations. First, as the number of citations may relate to the time since publication, some new “classic” articles may have been excluded as we analyzed only the most frequently cited articles and had no time limit. Second, we used only one limited database; different databases might result in a different list. Third, a number of factors could influence citation rates, including both journal and author self-citations; our analysis only assessed the information about citations but did not consider the other factors of it. Fourth, the value of contributions to the field cannot be quantified only by the number of citations. Finally, there have been so many studies on AD since it was first reported that the most-cited 100 articles may not be a true representation of the complete literature on this subject.

## Conclusions

Our analysis provides a historical perspective on the progress in AD research through a review of landmark papers on this condition, and indicates areas of research that require further attention and investigation. In addition, we analyzed the citation frequency of the articles published on AD as an indicator of study quality.

## Additional file

Additional file 1: Bibliometric information for the other 160 articles of the T100. (DOCX 50 kb)

## Abbreviations

AD: Aortic dissection
T100: The 100 most frequently cited articles
