# Supplementary material for: The 100 most-cited articles on aortic dissection
Source: BMC Cardiovasc Disord. 2017 Jan 17;17:30. doi: 10.1186/s12872-016-0426-9 (PMC5240425; doi:10.1186/s12872-016-0426-9)
Supplement: Additional file 1: — Bibliometric information for the other 160 articles of the T100. (DOCX 50 kb) [file 12872_2016_426_MOESM1_ESM.docx]

File1:Bibliometric information associated with the other 160 articles of the T100

| Rank | Author | Title | Journal | Year | Times cited | PMID |
| --- | --- | --- | --- | --- | --- | --- |
| 21 | Suzuki, T; et al | Clinical profiles and outcomes of acute type B aortic dissection in the current era: Lessons from the International Registry of Aortic Dissection (IRAD) | Circulation | 2003 | 219 |  |
| 22 | FANN, JI; et al | Surgical-management of aortic dissection during A 30-year period | Circulation | 1995 | 216 |  |
| 23 | Zhu, LM; et al | Mutations in myosin heavy chain 11 cause a syndrome associating thoracic aortic aneurysm/aortic dissection and patent ductus arteriosus | Nature Genetics | 2006 | 216 | 16444274 |
| 24 | SVENSSON, LG; et al | Dissection of the aorta and dissecting aortic-aneurysms - improving early and long-term surgical results | Circulation | 1990 | 215 |  |
| 25 | Khan, IA; et al | Clinical, diagnostic, and management perspectives of aortic dissection | Chest | 2002 | 213 | 12114376 |
| 26 | Nienaber, CA;et al | Aortic dissection: New frontiers in diagnosis and management - Part I: From etiology to diagnostic strategies | Circulation | 2003 | 213 | 12900496 |
| 27 | CRAWFORD, ES; et al | Surgical-treatment of aneurysm and or dissection of the ascending aorta, transverse aortic-arch, and ascending aorta and transverse aortic-arch - factors influencing survival in 717 patientS | Journal of Thoracic And Cardiovascular Surgery | 1989 | 205 | 2811404 |
| 28 | Tsai, Thomas T.; et al | Partial thrombosis of the false lumen in patients with acute type B aortic dissection | New England Journal of Medicine | 2007 | 203 | 17652650 |
| 29 | Nuenninghoff, DM; et al | Incidence and predictors of large-artery complication (aortic aneurysm, aortic dissection, and/or large-artery stenosis) in patients with giant cell arteritis - A population-based study over 50 years | Arthritis And Rheumatism | 2003 | 199 | 14674004 |
| 30 | Pannu, H; et al | Mutations in transforming growth factor-beta receptor type II cause familial thoracic aortic aneurysms and dissections | Circulation | 2005 | 192 | 16027248 |
| 31 | MILLER, DC; et al | Independent determinants of operative mortality for patients with aortic dissections | Circulation | 1984 | 187 |  |
| 32 | FROST, JJ; et al | Concurrent dissection and intracaval rupture of an abdominal aortic-aneurysm - ct findings | Journal of Computer Assisted Tomography | 1985 | 187 | 2982931 |
| 33 | Sommer, T; et al | Aortic dissection: A comparative study of diagnosis with spiral CT, multiplanar transesophageal echocardiography, and MR imaging | Radiology | 1996 | 185 | 8668776 |
| 34 | YAMADA, T; et al | Aortic dissection without intimal rupture - diagnosis with mr imaging and ct | Radiology | 1988 | 182 | 3393653 |
| 35 | Tsai, Thomas T.; et al | Long-term survival in patients presenting with type B acute aortic dissection - Insights from the International Registry of Acute Aortic Dissection | Circulation | 2006 | 181 | 17101856 |
| 36 | NIENABER, CA; et al | Diagnosis of thoracic aortic dissection - magnetic-resonance-imaging versus transesophageal echocardiography | Circulation | 1992 | 178 | 1735142 |
| 37 | Mehta, RH; et al | Predicting death in patients with acute type A aortic dissection | Circulation | 2002 | 174 | 11790701 |
| 38 | FANN, JI; et al | Treatment of patients with aortic dissection presenting with peripheral vascular complications | Annals of Surgery | 1990 | 172 | 2256762 |
| 39 | Olsson, Christian; et al | Thoracic aortic aneurysm and dissection - Increasing prevalence and improved outcomes reported in a nationwide population-based study of more than 14000 cases from 1987 to 2002 | Circulation | 2006 | 171 | 17145990 |
| 40 | Saraff, K; et al | Aortic dissection precedes formation of aneurysms and atherosclerosis in angiotensin II-infused, apolipoprotein E-deficient mice | Arteriosclerosis Thrombosis And Vascular Biology | 2003 | 170 | 12855482 |
| 41 | HAVERICH, A;et al | Acute and chronic aortic dissections - determinants of long-term outcome for operative survivors | Circulation | 1985 | 166 |  |
| 42 | Bernard, Y; et al | False lumen patency as a predictor of late outcome in aortic dissection | American Journal of Cardiology | 2001 | 159 | 11397357 |
| 43 | van de laar, et al | Mutations in SMAD3 cause a syndromic form of aortic aneurysms and dissections with early-onset osteoarthritis | Nature Genetics | 2011 | 159 | 21217753 |
| 44 | AMPARO, EG;et al | Aortic dissection - magnetic-resonance imaging | Radiology | 1985 | 159 | 3983390 |
| 45 | DOROGHAZI, RM; et al | Long-term survival of patients with treated aortic dissection | Journal of The American College of Cardiology | 1984 | 159 | 6707340 |
| 46 | GLOWER, DD; et al | Comparison of medical and surgical therapy for uncomplicated descending aortic dissection | Circulation | 1990 | 157 |  |
| 47 | Trimarchi, S; et al | Contemporary results of surgery in acute type A aortic dissection: The International Registry of Acute Aortic Dissection experience | Journal of Thoracic And Cardiovascular Surgery | 2005 | 155 | 15632832 |
| 48 | ELEFTERIADES, JA; et al | Long-term experience with descending aortic dissection - the complication-specific approach | Annals of Thoracic Surgery | 1992 | 151 | 1728218 |
| 49 | Fattori, Rossella; et al | Complicated Acute Type B Dissection: Is Surgery Still the Best Option? A Report From the International Registry of Acute Aortic Dissection | Jacc-Cardiovascular Interventions | 2008 | 147 | 19463336 |
| 50 | Pape, Linda A.; et al | Aortic diameter >= 5.5 cm is not a good predictor of type A aortic dissection - Observations from the international registry of acute aortic dissection (IRAD) | Circulation | 2007 | 146 | 17709637 |
| 51 | Januzzi, JL; et al | Characterizing the young patient with aortic dissection: Results from the international registry of aortic dissection (IRAD) | Journal of The American College of Cardiology | 2004 | 144 | 14975480 |
| 52 | Shiga, Toshiya; et al | Diagnostic accuracy of transesophageal echocardiography, helical computed tomography, and magnetic resonance imaging for suspected thoracic aortic dissection - Systematic review and meta-analysis | Archives of Internal Medicine | 2006 | 143 | 16831999 |
| 53 | ROBERTS, CS; et al | Dissection of the aorta associated with congenital-malformation of the aortic-valve | Journal of The American College of Cardiology | 1991 | 143 | 1993792 |
| 54 | Immer, FF; et al | Aortic dissection in pregnancy: Analysis of risk factors and outcome | Annals of Thoracic Surgery | 2003 | 142 | 12842575 |
| 55 | Clouse, WD;et al | Acute aortic dissection: Population-based incidence compared with degenerative aortic aneurysm rupture | Mayo Clinic Proceedings | 2004 | 138 | 14959911 |
| 57 | Slonim, SM; et al | Aortic dissection: Percutaneous management of ischemic complications with endovascular stents and balloon fenestration | Journal of Vascular Surgery | 1996 | 138 | 8637101 |
| 58 | Golledge, Jonathan;et al | Acute aortic dissection | Lancet | 2008 | 135 | 18603160 |
| 59 | BALLAL, RS;et al | Usefulness of transesophageal echocardiography in assessment of aortic dissection | Circulation | 1991 | 135 | 1934367 |
| 60 | Kato, M; et al | New graft-implanting method for thoracic aortic aneurysm or dissection with a stented graft | Circulation | 1996 | 134 |  |
| 61 | KAZEROONI, EA;et al | Penetrating atherosclerotic ulcers of the descending thoracic aorta - evaluation with ct and distinction from aortic dissection | Radiology | 1992 | 133 | 1584933 |
| 62 | Milewicz, Dianna M.; et al | Genetic basis of thoracic aortic aneurysms and dissections: Focus on smooth muscle cell contractile dysfunction | Annual Review of Genomics And Human Genetics | 2008 | 133 | 18544034 |
| 63 | CRAWFORD, ES; et al | Aortic dissection and dissecting aortic-aneurysms | Annals of Surgery | 1988 | 132 | 3421752 |
| 64 | Tieu, Brian C.;et al | An adventitial IL-6/MCP1 amplification loop accelerates macrophage-mediated vascular inflammation leading to aortic dissection in mice | Journal of Clinical Investigation | 2009 | 130 | 19920349 |
| 65 | Nienaber, CA; et al | Aortic dissection: new frontiers in diagnosis and management - Part II: Therapeutic management and follow-up | Circulation | 2003 | 125 | 12912795 |
| 66 | GUILMET, D; et al | Use of biological glue in acute aortic dissection - preliminary clinical results with a new surgical technique | Journal of Thoracic And Cardiovascular Surgery | 1979 | 125 | 423584 |
| 67 | Guo, DC; et al | Familial thoracic aortic aneurysms and dissections - Genetic heterogeneity with a major locus mapping to 5q13-14 | Circulation | 2001 | 123 | 11369686 |
| 68 | Umana, JP; et al | Is medical therapy still the optimal treatment strategy for patients with acute type B aortic dissections? | Journal of Thoracic And Cardiovascular Surgery | 2002 | 120 | 12407372 |
| 69 | Mehta, RH; et al | Acute type A aortic dissection in the elderly: Clinical characteristics, management, and outcomes in the current era | Journal of The American College of Cardiology | 2002 | 119 | 12204498 |
| 70 | Svensson, LG; et al | Intimal tear without hematoma - An important variant of aortic dissection that can elude current imaging techniques | Circulation | 1999 | 119 | 10077517 |
| 71 | FRANCKE, U; et al | A gly1127ser mutation in an egf-like domain of the fibrillin-1 gene is a risk factor for ascending aortic-aneurysm and dissection | American Journal of Human Genetics | 1995 | 119 | 7762551 |
| 72 | Keren, A;et al | Accuracy of biplane and multiplane transesophageal echocardiography in diagnosis of typical acute aortic dissection and intramural hematoma | Journal of The American College of Cardiology | 1996 | 117 | 8772749 |
| 73 | Elefteriades, JA; et al | Management of descending aortic dissection | Annals of Thoracic Surgery | 1999 | 116 | 10391358 |
| 74 | von Kodolitsch, Y; et al | Clinical prediction of acute aortic dissection | Archives of Internal Medicine | 2000 | 115 | 11041906 |
| 75 | LIN, AE;et al | Aortic dilation, dissection, and rupture in patients with turner syndrome | Journal of Pediatrics | 1986 | 115 | 3772661 |
| 76 | CRAWFORD, ES | The diagnosis and management of aortic dissection | Jama-Journal of The American Medical Association | 1990 | 114 | 2232021 |
| 77 | Albornoz, Gonzalo; et al | Familial thoracic aortic aneurysms and dissections - Incidence, modes of inheritance, and phenotypic patterns | Annals of Thoracic Surgery | 2006 | 114 | 16996941 |
| 78 | BACHET, JE; et al | Aortic dissection - prevalence, cause, and results of late reoperations | Journal of Thoracic And Cardiovascular Surgery | 1994 | 114 | 8041167 |
| 79 | ERBEL, R; et al | Detection of aortic dissection by transesophageal echocardiography | British Heart Journal | 1987 | 114 | 3620241 |
| 80 | NANDA, NC;et al | Diagnosis of aortic root dissection by echocardiography | Circulation | 1973 | 114 | 4726233 |
| 81 | Vilacosta, I; et al | Natural history and serial morphology of aortic intramural hematoma: A novel variant of aortic dissection | American Heart Journal | 1997 | 112 | 9327708 |
| 82 | Nienaber, CA; et al | INvestigation of STEnt grafts in patients with type B Aortic Dissection: Design of the INSTEAD trial - a prospective, multicenter, European randomized trial | American Heart Journal | 2005 | 111 | 15990739 |
| 83 | Pretre, R; et al | Aortic dissection | Lancet | 1997 | 110 | 9164331 |
| 84 | Rampoldi, Vincenzo; et al | Simple risk models to predict surgical mortality in acute type A aortic dissection: The International Registry of Acute Aortic Dissection score | Annals of Thoracic Surgery | 2007 | 110 | 17184630 |
| 85 | GLOWER, DD; et al | Management and long-term outcome of aortic dissection | Annals of Surgery | 1991 | 110 | 2064469 |
| 86 | Mehta, RH; et al | Chronobiological patterns of acute aortic dissection | Circulation | 2002 | 109 | 12196337 |
| 87 | Slonim, SM;et al | Percutaneous balloon fenestration and stenting for life-threatening ischemic complications in patients with acute aortic dissection | Journal of Thoracic And Cardiovascular Surgery | 1999 | 107 | 10343260 |
| 88 | Matura, Lea Ann;et al | Aortic dilatation and dissection in Turner syndrome | Circulation | 2007 | 107 | 17875973 |
| 89 | Nienaber, CA; et al | Gender-related differences in acute aortic dissection | Circulation | 2004 | 106 | 15197151 |
| 90 | Sabik, JF; et al | Long-term effectiveness of operations for ascending aortic dissections | Journal of Thoracic And Cardiovascular Surgery | 2000 | 106 | 10788816 |
| 91 | Eggebrecht, H; et al | Value of plasma fibrin D-dimers for detection of acute aortic dissection | Journal of The American College of Cardiology | 2004 | 106 | 15312863 |
| 92 | GEISINGER, MA;et al | Thoracic aortic dissections - magnetic-resonance imaging | Radiology | 1985 | 106 | 3983391 |
| 93 | MASUDA, Y;et al | Prognosis of patients with medically treated aortic dissections | Circulation | 1991 | 105 |  |
| 94 | Marui, A;et al | Toward the best treatment for uncomplicated patients with type B acute aortic dissection - A consideration for sound surgical indication | Circulation | 1999 | 104 |  |
| 95 | Tsai, T. T.; et al | Acute Aortic Dissection: Perspectives from the International Registry of Acute Aortic Dissection (IRAD) | European Journal of Vascular And Endovascular Surgery | 2009 | 103 | 19097813 |
| 96 | Halstead, James C.; et al | The fate of the distal aorta after repair of acute type A aortic dissection | Journal of Thoracic And Cardiovascular Surgery | 2007 | 103 | 17198797 |
| 97 | Klompas, M | Does this patient have an acute thoracic aortic dissection? | Jama-Journal of The American Medical Association | 2002 | 103 | 11980527 |
| 98 | Chuter, TAM; et al | Modular branched stent graft for endovascular repair of aortic arch aneurysm and dissection | Journal of Vascular Surgery | 2003 | 102 | 14560246 |
| 99 | VICTOR, MF; et al | Two-dimensional echocardiographic diagnosis of aortic dissection | American Journal of Cardiology | 1981 | 102 | 7304463 |
| 100 | Nienaber, Christoph A.;et al | Provisional extension to induce complete attachment after stent-graft placement in type B aortic dissection: The PETTICOAT concept | Journal of Endovascular Therapy | 2006 | 98 | 17154712 |
| 101 | Weber, T; et al | D-dimer in acute aortic dissection | Chest | 2003 | 97 | 12740250 |
| 102 | David, TE; et al | Surgery for acute type A aortic dissection | Annals of Thoracic Surgery | 1999 | 96 | 10391357 |
| 103 | Akutsu, K; et al | Effects of the patent false lumen on the long-term outcome Iq of type B acute aortic dissection | European Journal of Cardio-Thoracic Surgery | 2004 | 96 | 15296897 |
| 104 | Gonzalez-Gay, MA; et al | Aortic aneurysm and dissection in patients with biopsy-proven giant cell arteritis from northwestern Spain - A population-based study | Medicine | 2004 | 96 | 15525845 |
| 105 | KERSTINGSOMMERHOFF, BA; et al | AORTIC DISSECTION - sensitivity and specificity of mr imaging | Radiology | 1988 | 95 | 3340758 |
| 106 | He, RM; et al | Characterization of the inflammatory and apoptotic cells in the aortas of patients with ascending thoracic aortic aneurysms and dissections | Journal of Thoracic And Cardiovascular Surgery | 2006 | 93 | 16515922 |
| 107 | Koullias, GJ; et al | Increased tissue microarray matrix metalloproteinase expression favors proteolysis in thoracic aortic aneurysms and dissections | Annals of Thoracic Surgery | 2004 | 93 | 15561045 |
| 108 | Hansen, CJ; et al | Complications of endovascular repair of high-risk and emergent descending thoracic aortic aneurysms and dissections | Journal of Vascular Surgery | 2004 | 93 | 15297815 |
| 109 | WILLIAMS, DM; et al | Relief of mesenteric ischemia in type-iii aortic dissection with percutaneous fenestration of the aortic septum | Radiology | 1990 | 93 | 2136956 |
| 110 | White, RA; et al | Endovascular exclusion of descending thoracic aortic aneurysms and chronic dissections: Initial clinical results with the AneuRx device | Journal of Vascular Surgery | 2001 | 92 | 11331830 |
| 111 | Kazui, T;et al | Extended total arch replacement for acute type a aortic dissection: Experience with seventy patients | Journal of Thoracic And Cardiovascular Surgery | 2000 | 91 | 10694617 |
| 112 | Neri, E; et al | Axillary artery cannulation in type A aortic dissection operations | Journal of Thoracic And Cardiovascular Surgery | 1999 | 91 | 10425006 |
| 113 | Hasham, SN; et al | Mapping a locus for familial thoracic aortic aneurysms and dissections (TAAD2) to 3p24-25 | Circulation | 2003 | 90 | 12821554 |
| 114 | Song, Jong-Min;et al | Long-term predictors of descending aorta Aneurysmal change in patients with aortic dissection | Journal of The American College of Cardiology | 2007 | 90 | 17707186 |
| 115 | Muhs, Bart E.; et al | Anatomic factors associated with acute endograft collapse after Gore TAG treatment of thoracic aortic dissection or traumatic rupture | Journal of Vascular Surgery | 2007 | 90 | 17306949 |
| 116 | MOHRKAHALY, S; et al | Ambulatory follow-up of aortic dissection by trans-esophageal two-dimensional and color-coded doppler echocardiography | Circulation | 1989 | 90 | 2736753 |
| 117 | Song, JK; et al | Different clinical features of aortic intramural hematoma versus dissection involving the ascending aorta | Journal of The American College Of Cardiology | 2001 | 89 | 11345372 |
| 118 | Moore, AG; et al | Choice of computed tomography, transesophageal echocardiography, magnetic resonance imaging, and aortography in acute aortic dissection: International Registry of Acute Aortic Dissection (IRAD) | American Journal of Cardiology | 2002 | 89 | 12008187 |
| 119 | Kazui, T; et al | Role of biologic glue repair of proximal aortic dissection in the development of early and midterm redissection of the aortic root | Annals of Thoracic Surgery | 2001 | 89 | 11515890 |
| 120 | Szeto, Wilson Y.; et al | Results of a new surgical paradigm: Endovascular repair for acute complicated type B aortic dissection | Annals of Thoracic Surgery | 2008 | 88 | 18573403 |
| 121 | Bergeron, P.;et al | Great vessel management for endovascular exclusion of aortic arch aneurysms and dissections | European Journal of Vascular And Endovascular Surgery | 2006 | 88 | 16520069 |
| 122 | Gravholt, Claus Hojbjerg; et al | Clinical and epidemiological description of aortic dissection in Turner's syndrome | Cardiology In The Young | 2006 | 88 | 16984695 |
| 123 | OGARA, PT; et al | Acute aortic dissection and its variants - toward a common diagnostic and therapeutic approach | Circulation | 1995 | 88 | 7664413 |
| 124 | EGAN, TJ; et al | Computed-tomography in the diagnosis of aortic-aneurysm dissection or traumatic injury | Radiology | 1980 | 88 | 7384488 |
| 125 | Wang, YB; et al | VKORC1 haplotypes are associated with arterial vascular diseases (stroke, coronary heart disease, and aortic dissection) | Circulation | 2006 | 87 | 16549638 |
| 126 | Eggebrecht, H;et al | Endovascular stent-graft treatment of aortic dissection: determinants of post-interventional outcome | European Heart Journal | 2005 | 86 | 15673541 |
| 127 | KATO, M; et al | Determining surgical indications for acute type-b dissection based on enlargement of aortic diameter during the chronic phase | Circulation | 1995 | 86 |  |
| 128 | Suzuki, Toru; et al | Diagnosis of Acute Aortic Dissection by D-Dimer The International Registry of Acute Aortic Dissection Substudy on Biomarkers (IRAD-Bio) Experience | Circulation | 2009 | 85 | 19433758 |
| 129 | Schoder, Maria; et al | Endovascular repair of acute type B aortic dissection: Long-term follow-up of true and false lumen diameter changes | Annals of Thoracic Surgery | 2007 | 85 | 17307460 |
| 130 | FANN, JI; et al | Preservation of aortic-valve in type-a aortic dissection complicated by aortic regurgitation | Journal of Thoracic And Cardiovascular Surgery | 1991 | 84 | 2072730 |
| 131 | Lauterbach, SR; et al | Contemporary management of aortic branch compromise resulting from acute aortic dissection | Journal of Vascular Surgery | 2001 | 83 | 11389416 |
| 132 | Querleu, D; et al | Extraperitoneal endosurgical aortic and common iliac dissection in the staging of bulky or advanced cervical carcinomas | Cancer | 2000 | 83 | 10760766 |
| 133 | Bachet, J;et al | Surgery for acute type A aortic dissection: The Hopital Foch experience (1977-1998) | Annals of Thoracic Surgery | 1999 | 83 | 10391359 |
| 134 | RIZZO, RJ; et al | Rapid noninvasive diagnosis and surgical repair of acute ascending aortic dissection - improved survival with less angiography | Journal of Thoracic And Cardiovascular Surgery | 1994 | 82 | 8078350 |
| 135 | WALKER, PJ;et al | The use of endovascular techniques for the treatment of complications of aortic dissection | Journal of Vascular Surgery | 1993 | 82 | 8264033 |
| 136 | Yoshida, S; et al | Thoracic involvement of type A aortic dissection and intramural hematoma: Diagnostic accuracy - Comparison of emergency helical CT and surgical findings | Radiology | 2003 | 81 | 12819341 |
| 137 | Mitchell, RS; et al | First international summit on thoracic aortic endografting: Roundtable on thoracic aortic dissection as an indication for endografting | Journal of Endovascular Therapy | 2002 | 81 |  |
| 138 | EARNEST, F;et al | Roentgenographic findings in thoracic aortic dissection | Mayo Clinic Proceedings | 1979 | 81 | 759737 |
| 139 | Regalado, Ellen S.; et al | Exome Sequencing Identifies SMAD3 Mutations as a Cause of Familial Thoracic Aortic Aneurysm and Dissection With Intracranial and Other Arterial Aneurysms | Circulation Research | 2011 | 81 | 21778426 |
| 140 | Kirsch, M; et al | Risk factor analysis for proximal and distal reoperations after surgery for acute type A aortic dissection | Journal of Thoracic And Cardiovascular Surgery | 2002 | 81 | 11828292 |
| 141 | BROWN, OR; et al | Echocardiographic criteria for aortic root dissection | American Journal of Cardiology | 1975 | 81 | 1146693 |
| 142 | Czermak, BV; et al | Treatment of Stanford type B aortic dissection with stent-grafts: Preliminary results | Radiology | 2000 | 80 | 11058658 |
| 143 | Biddinger, A; et al | Familial thoracic aortic dilatations and dissections: A case control study | Journal of Vascular Surgery | 1997 | 80 | 9081132 |
| 144 | Zierer, Andreas; et al | Aortic enlargement and late reoperation after repair of acute type A aortic dissection | Annals of Thoracic Surgery | 2007 | 79 | 17643619 |
| 145 | Eggebrecht, Holger;et al | Retrograde Ascending Aortic Dissection During or After Thoracic Aortic Stent Graft Placement Insight From the European Registry on Endovascular Aortic Repair Complications | Circulation | 2009 | 79 | 19752379 |
| 146 | Pereles, FS; et al | Thoracic aortic dissection and aneurysm: Evaluation with nonenhanced true FISP MR angiography in less than 4 minutes | Radiology | 2002 | 78 | 11930077 |
| 147 | Kusagawa, H; et al | Changes in false lumen after transluminal stent-graft placement in aortic dissections - Six years' experience | Circulation | 2005 | 78 | 15927978 |
| 148 | Schor, JS; et al | Selective management of acute type B aortic dissection: Long term follow-up | Annals of Thoracic Surgery | 1996 | 78 | 8633937 |
| 149 | HEIBERG, E; et al | CT findings in thoracic aortic dissection | American Journal of Roentgenology | 1981 | 78 | 6779559 |
| 150 | Svensson, LG; et al | Relationship of aortic cross-sectional area to height ratio and the risk of aortic dissection in patients with bicuspid aortic valves | Journal of Thoracic And Cardiovascular Surgery | 2003 | 78 | 14502185 |
| 151 | Palma, JH; et al | Self-expandable aortic stent-grafts for treatment of descending aortic dissections | Annals of Thoracic Surgery | 2002 | 77 | 11996254 |
| 152 | CAMBRIA, RP; et al | Vascular complications associated with spontaneous aortic dissection | Journal of Vascular Surgery | 1988 | 77 | 3276932 |
| 153 | BURCHELL, HB | Aortic dissection (dissecting hematoma - dissecting aneurysm of the aorta) | Circulation | 1955 | 77 | 13270364 |
| 154 | HAYASHI, K; et al | Aortographic analysis of aortic dissection | American Journal of Roentgenology | 1974 | 77 | 4458454 |
| 155 | Sebastia, C; et al | Aortic dissection: Diagnosis and follow-up with helical CT | Radiographics | 1999 | 76 | 9925391 |
| 156 | Boileau, Catherine; et al | TGFB2 mutations cause familial thoracic aortic aneurysms and dissections associated with mild systemic features of Marfan syndrome | Nature Genetics | 2012 | 76 | 22772371 |
| 157 | Hsue, PY; et al | Acute aortic dissection related to crack cocaine | Circulation | 2002 | 76 | 11927528 |
| 158 | BACHET, J; et al | Replacement of the transverse aortic-arch during emergency operations for type-a acute aortic dissection - report of 26 cases | Journal of Thoracic And Cardiovascular Surgery | 1988 | 76 | 3269219 |
| 159 | LARDE, D; et al | Computed-tomography of aortic dissection | Radiology | 1980 | 76 | 7384489 |
| 160 | Boeckler, Dittmar; et al | Complications after endovascular repair of acute symptomatic and chronic expanding Stanford type B aortic dissections | Journal of Thoracic And Cardiovascular Surgery | 2006 | 74 | 16872963 |
| 161 | Coselli, JS;et al | Paraplegia after thoracoabdominal aortic aneurysm repair: Is dissection a risk factor? | Annals of Thoracic Surgery | 1997 | 74 | 8993237 |
| 162 | Batra, P; et al | Pitfalls in the diagnosis of thoracic aortic dissection at CT angiography | Radiographics | 2000 | 74 | 10715333 |
| 163 | GUTHANER, DF; et al | Fate of the false lumen following surgical repair of aortic dissections - angiographic study | Radiology | 1979 | 74 | 382246 |
| 164 | Dong, Zhi Hui; et al | Retrograde Type A Aortic Dissection After Endovascular Stent Graft Placement for Treatment of Type B Dissection | Circulation | 2009 | 73 | 19171859 |
| 165 | Nuenninghoff, DM; et al | Mortality of large-artery complication (Aortic aneurysm, aortic dissection, and/or large-artery stenosis) in patients with giant cell arteritis - A population-based study over 50 years | Arthritis and Rheumatism | 2003 | 73 | 14674005 |
| 166 | BACHET, J; et al | Surgery of type-a acute aortic dissection with gelatin-resorcine-formol biological glue - a 12-year experience | Journal of Cardiovascular Surgery | 1990 | 73 | 2370256 |
| 167 | Ohlmann, P;et al | Diagnostic and prognostic value of circulating D-Dimers in patients with acute aortic dissection | Critical Care Medicine | 2006 | 72 | 16557157 |
| 168 | Nienaber, Christoph A.;et al | Endovascular Repair of Type B Aortic Dissection Long-term Results of the Randomized Investigation of Stent Grafts in Aortic Dissection Trial | Circulation-Cardiovascular Interventions | 2013 | 72 | 23922146 |
| 169 | EAGLE, KA; et al | Spectrum of conditions initially suggesting acute aortic dissection but with negative aortograms | American Journal of Cardiology | 1986 | 71 | 3946223 |
| 170 | Suzuki, T; et al | Novel biochemical diagnostic method for aortic dissection - Results of a prospective study using an immunoassay of smooth muscle myosin heavy chain | Circulation | 1996 | 71 | 8653847 |
| 171 | Yetman, AT; et al | Long-term outcome in patients with Marfan syndrome: Is aortic dissection the only cause of sudden death? | Journal of The American College of Cardiology | 2003 | 70 | 12535830 |
| 172 | Ledbetter, S;et al | Helical (spiral) CT in the evaluation of emergent thoracic aortic syndromes - Traumatic aortic rupture, aortic aneurysm, aortic dissection, intramural hematoma, and penetrating atherosclerotic ulcer | Radiologic Clinics of North America | 1999 | 70 | 10361547 |
| 173 | STILL, RJ; et al | Intraoperative aortic dissection | Annals of Thoracic Surgery | 1992 | 70 | 1540051 |
| 174 | Pansini, S; et al | Early and late risk factors in surgical treatment of acute type A aortic dissection | Annals of Thoracic Surgery | 1998 | 69 | 9768930 |
| 175 | Liu, ZG; et al | Should the all elephant trunk" be skeletonized? Total arch replacement combined with stented elephant trunk implantation for Stanford type A aortic dissection | Journal of Thoracic And Cardiovascular Surgery | 2006 | 68 | 16399301 |
| 176 | Hayter, RG; et al | Suspected aortic dissection and other aortic disorders: Multi-detector row CT in 373 cases in the emergency setting | Radiology | 2006 | 68 | 16452396 |
| 177 | Macura, KJ; et al | Pathogenesis in acute aortic syndromes: Aortic dissection, intramural hematoma, and penetrating atherosclerotic aortic ulcer | American Journal of Roentgenology | 2003 | 68 | 12876003 |
| 178 | Onitsuka, S; et al | Long-term outcome and prognostic predictors of medically treated acute type B aortic dissections | Annals of Thoracic Surgery | 2004 | 68 | 15464484 |
| 179 | BANSAL, RC; et al | Frequency and explanation of false-negative diagnosis of aortic dissection by aortography and transesophageal echocardiography | Journal of The American College of Cardiology | 1995 | 68 | 7722139 |
| 180 | VASILE, N; et al | Computed-tomography of thoracic aortic dissection - accuracy and pitfalls | Journal of Computer Assisted Tomography | 1986 | 68 | 3950147 |
